# Burnout and Workforce Withdrawal Intentions Among Psychiatry Inpatient Staff

**DOI:** 10.1192/bjo.2026.11649

**Published:** 2026-06-30

**Authors:** Mohamed Jalloh, Samukeliso Fundira, Vasudevan Krishnan

**Affiliations:** 1 St Georges Hospital, Midlands Partnership University NHS Foundation Trust, Stafford, United Kingdom; 2 Royal Stoke University Hospital, University Hospitals North Midlands, Stoke-On-Trent, United Kingdom

## Abstract

**Aims:**

Burnout is a recognised risk factor for workforce attrition in healthcare. In psychiatry in patient services, where retention pressures are high, understanding the relationship between burnout and staff intentions to leave or reduce working hours is critical for service sustainability. There we examine the association between burnout and workforce withdrawal intentions among psychiatry inpatient staff.

**Methods:**

An anonymous cross-sectional survey was completed by 115 inpatient psychiatry staff. Burnout was measured using a validated composite score. Workforce withdrawal intentions were assessed using two Likert-scale items measuring likelihood of leaving the current role and likelihood of reducing working hours within the next 12 months due to work-related stressors. Multivariable linear regression models adjusted for professional role group, years working in psychiatry, and work pattern. All statistical analyses were performed using IBM SPSS Statistics.

**Results:**

Higher burnout was strongly associated with increased intention to leave the current role within the next 12 months (adjusted B=0.94, p < 0.001), with the model explaining 47% of variance in turnover intention (adjusted R²=0.47). Burnout was also independently associated with intention to reduce working hours (adjusted B=0.62, p < 0.001; adjusted R²=0.21). Staff characteristics, including professional role, years of experience, and work pattern, were not significant predictors of workforce withdrawal intentions.

**Conclusion:**

Burnout is a powerful predictor of workforce withdrawal intentions among psychiatry inpatient staff. Addressing burnout may be critical to improving staff retention and maintaining workforce capacity in inpatient psychiatric services.

